# Unexpectedly High Prevalence of Hepatitis C Virus Infection, Southern Laos

**DOI:** 10.3201/eid2801.211307

**Published:** 2022-01

**Authors:** Antony P. Black, Vilaysone Khounvisith, Kinnaly Xaydalasouk, Kong Sayasinh, Aurelie Sausy, Claude P. Muller, Judith M. Hübschen

**Affiliations:** Institut Pasteur du Laos, Vientiane, Laos (A.P. Black, V. Khounvisith, K. Xaydalasouk);; Saravan Provincial Hospital, Saravan, Laos (K. Sayasinh);; Luxembourg Institute of Health, Esch-sur-Alzette, Luxembourg (A. Sausy, C.P. Muller, J.M. Hübschen)

**Keywords:** Hepatitis C virus, antibodies, Laos, serologic tests, sequencing, Lao People’s Democratic Republic, Laos, viruses

## Abstract

During 2017–2019, a total of 88/753 (11.7%) of patients 5–90 years of age in hospitals in Saravan Province, Laos, were seropositive for hepatitis C virus antibodies. Viral RNA was found in 44 samples. Sequencing showed high diversity within genotype 6. We recommend exposure-risk investigations and targeted testing and treatment.

Hepatitis C virus (HCV) infection carries high risk for progression to chronic status and liver complications, such as cirrhosis and cancer. Transmission usually occurs through blood (e.g., during medical procedures, blood transfusions, tattooing, or intravenous drug use). Because those who clear the virus remain HCV antibody positive, testing for viral RNA is essential for diagnosis of chronic infection (*1*).

We conducted a cross-sectional, hospital-based study during May 2017–March 2019 to determine seroprevalence and genotyping of HCV in Saravan Province in southern Laos. Saravan Province has a population of ≈400,000 distributed over 8 districts, 2 bordering Vietnam to the east and 2 bordering Thailand to the west. In 2017, only 8.5% of men and 6.9% of women had health insurance; 36.8% of the provincial population was in the poorest wealth index quartile; 17.8% of households had no electricity; and only 54.3% of men and 44.7% of women were literate, the lowest literacy rates in Laos (*2*).

We nonrandomly selected 753 participants from a larger study (Appendix) (*3*); participants were persons >5 years of age who were recruited for the larger study while seeking care at the provincial hospital or 1 of 3 district hospitals. Overall, 11.7% (88) participants were HCV antibody seropositive, compared with <2% in previous studies in Laos (*4*,*5*) (Figure; Appendix). Only 2 seropositive patients were at the hospital for hepatitis-related reasons; HCV seroprevalence was not significantly different regardless of whether or not participants sought care for reasons associated with hepatitis. After multivariate analysis, those >30 years of age had much higher seroprevalence (70/350, 20%) than those <30 years of age (18/403, 4.5%; odds ratio [OR] 4.2; p<0.001). This higher seroprevalence indicates either that older adults are at higher risk for exposure or that the older adults were infected some time ago, during childhood or early adulthood. Participants who practice Animism had a slightly higher seroprevalence (81/495; 16.4%) than followers of Buddhism or other faiths (7/258, 3.0%; OR 5.1; p = 0.02), and married participants had slightly higher seroprevalence (81/485, 16.7%) than single participants (7/268, 2.6%; OR 2.7; p = 0.04), although the associated risk factors are unknown (Table; Appendix).

**Figure:**
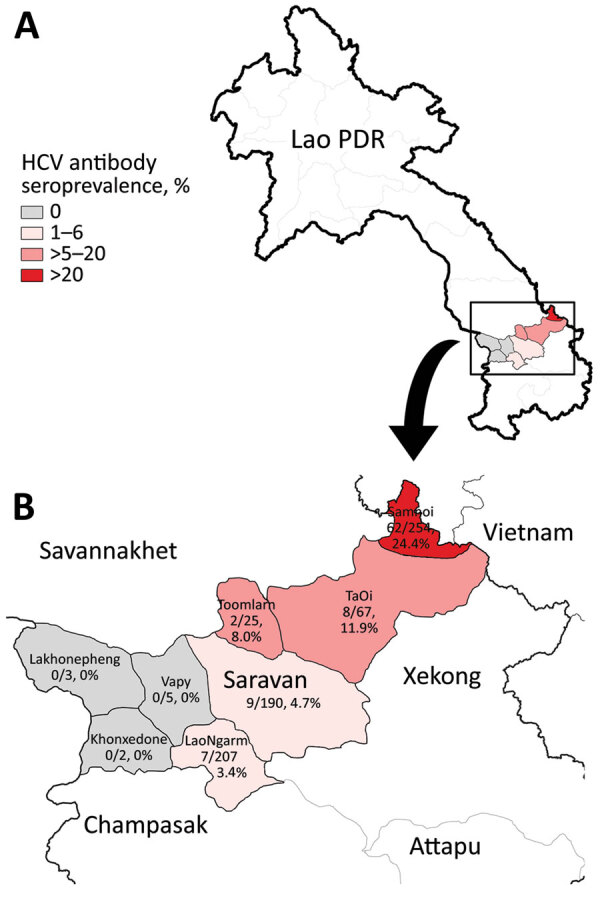
HCV seroprevalence, Laos, May 2017–March 2019. A) Location of Saravan Province; B) districts of Saravan Province. Colors represent seroprevalence levels. HCV, hepatitis C virus

**Table:** Bivariate and multivariate analysis of HCV antibody positive cases, Laos, May 2017–March 2019*

Variable	Positive no./total no. (%)	Bivariate			Multivariate	
OR (95% CI)	p value	OR (95% CI)	p value
Sex						
F	42/417 (10.1)	Referent	NA		Referent	NA
M	46/336 (13.7)	1.4 (0.9–2.2)	0.126		1.5 (0.9–2.6)	0.08
Age group, y						
<30	18/403 (4.5)	Referent			Referent	NA
>30	70/350 (20)	5.3 (3.1–9.2)	<0.001		4.2 (2.1–8.5)	<0.001
District†						
Lao Ngarm	7/207 (3.4)	Referent			NA	NA
Saravan	9/190 (4.7)	1.4 (0.5–3.9)	0.495		NA	NA
Samuoi‡	62/254 (24.4)	9.2 (4.1–20.7)	<0.001		NS	NS
Ta Oi	8/67 (11.9)	3.9 (1.3–11.1)	0.012		NS	NS
Toomlarn	2/25 (8)	2.5 (0.5–12.7)	0.274		NA	NA
Vapy	0/5 (0)	NA	NA		NA	NA
Khongxedone	0/2 (0)	NA	NA		NA	NA
Lakhonepheng	0/3 (0)	NA	NA		NA	NA
Marital status						
Single	7/268 (2.6)	Referent	NA		Referent	NA
Married or other	81/485 (16.7)	7.5 (3.4–16.4)	<0.001		2.7 (1.0–7.3)	0.04
Occupation						
Student or other	7/231 (3)	Referent	NA		NA	NA
Employee	14/67 (20.9)	8.5 (3.3–21.9)	<0.001		NS	NS
Farmer	67/455 (14.7)	5.5 (2.5–12.2)	<0.001		NS	NS
Ethnicity						
Non-Pako	22/488(4.5)	Referent	NA		Referent	NA
Pako	66/265 (24.9)	7 (4.2–11.7)	<0.001		5.1 (2.7–9.7)	<0.001
Religion						
Buddhist or other	7/258 (2.7)	Referent	NA		Referent	NA
Animism	81/495 (16.4)	7 (3.2–15.4)	<0.001		3 (1.2–7.6)	0.02
Place of birth						
At hospital or unknown	57/440 (12.9)	Referent	NA		NA	NA
At home	31/313 (9.9)	0.7 (0.4–1.2)	0.2		NA	NA
Diagnosis						
Hepatitis non-related or unknown	86/702 (12.3)	Referent	NA		Referent	NA
Related to hepatitis	2/51 (3.9)	0.2 (0.1–1.2)	0.09		0.2 (0.1–1.1)	0.07

*NA, not applicable; NS, not significant; OR, odds ratio. †District was not included in the multivariate analysis because it reduced the power of the model. ‡Samuoi district correlated strongly with Pako ethnicity (Pearson correlation coefficient, R = 0.48, p<0.001).

Whether the observed west–east increase in seroprevalence is related to the proximity of Samuoi district (24.4% anti-HCV seropositive) to the Vietnam border remains unclear (Figure; Appendix). Although HCV seroprevalence in Quang Tri, a bordering province in Vietnam, has been reported to be <1% (*6*), much higher rates were found in different groups at high risk in Vietnam, such as intravenous drug users (IDU) and men who have sex with men (MSM) (*7*). We could find no reported link between the Samuoi district population and the IDU or MSM communities in Vietnam, although this link remains possible. 

Seroprevalence was significantly higher among the Pako ethnic group (66/265, 24.9% vs. 22/488, 4.5%; OR 5.1; p<0.001), which makes up most of the population in Samuoi district but not elsewhere. The Pako practice nonsterile teeth filing and lacquering during early adolescence with shared equipment and associated bleeding, although this practice is in decline. Pako do not often practice tattooing, but the women have ear piercings, which could be another source of infection. Other risk factors, such as blood transfusions and practices of MSM and IDU, are thought to be rare in this population, but nonsterile injection of traditional medicine might occur (*8*; A. Sernsarae, Samuoi District Health Office, pers. comm., 2020 Jul 23).

Only 44 of the samples we tested were positive for HCV RNA. The relatively low rate of chronic infection could indicate exposure early in life; persons infected at <25 years of age are thought to have much lower risk for chronic infection (*1*). A substantial proportion of children in our study were also infected, either by mother-to-child transmission or through the same routes as the adults in the study.

We obtained sequence data for 39 samples. All belonged to genotype 6 (Appendix). The sequence diversity does not suggest any recent large-scale transmission events, because no identical sequences were obtained, and the many genetically diverse clusters even in the same district (Samuoi) might indicate different infection sources. However, we cannot rule out a more distant large-scale transmission event. The new strains added to the genetic diversity of genotype 6 viruses found in a previous study from central and northern Laos provinces (*5*); this increased diversity has potential consequences for the use of commercial assays (*9*) and treatment strategies (*10*).

The high rates of death and illness associated with chronic HCV infection suggest that a large proportion of the Saravan population will experience liver-related complications in the future. Despite a reduction in costs of direct-acting antiviral drugs, access to testing and treatment remains low in Laos. An in-depth case-control study to determine sources of infection and associated risk factors is warranted. Furthermore, evaluations of infection prevention, screening, and control measures in healthcare facilities and blood banks, as well as the general population, are needed.

Appendix Additional information about unexpectedly high prevalence of hepatitis C virus infection, southern Laos.
