# Supplementary material for: Unexpectedly High Prevalence of Hepatitis C Virus Infection, Southern Laos
Source: Emerg Infect Dis. 2022 Jan;28(1):256–9. doi: 10.3201/eid2801.211307 (PMC8714196; doi:10.3201/eid2801.211307)
Supplement: Appendix — Additional information about unexpectedly high prevalence of hepatitis C virus infection, southern Laos. [file 21-1307-Techapp-s1.pdf]

Article DOI: https://doi.org/10.3201/eid2801.211307

Unexpectedly High Prevalence of Hepatitis C Virus Infection, Southern Laos

Appendix

Participant Recruitment

Because of limited serum sample volume and reagent availability, a subset of 753 samples from a larger study on infectious diseases involving 2,500 participants (*1*) were tested for hepatitis C virus (HCV) antibodies. Initial testing indicated a high seroprevalence in Samoui district, Saravan Province, Lao People's Democratic Republic. Therefore all 254 samples from Samuoi district were selected, and an additional 499 samples were randomly selected from other districts.

Participants >5 years of age were recruited while seeking care at the provincial or one of 3 district hospitals. Both inpatients and outpatients were asked to participate in the study by healthcare staff and gave informed consent after reading the information sheet. In the case of participants unable to read Lao language, the healthcare workers read the form, and for participants unable to sign, a fingerprint was taken with the signature of a witness. The parents or guardians gave consent for children <18 years of age. Patients with jaundice or hepatitis-related complications were not excluded from participation. The study was approved by the Lao National Research Ethics Committee (ref. no. 005/2018 NECHR), and the data have been reported to the Lao Ministry of Health.

Questionnaire and Serologic Testing

After collecting demographic information (age, sex, hometown, place of birth, religion, occupation, ethnicity, reason for hospitalization, marital status), 5 mL blood specimens were taken from participants. The blood was allowed to clot, and serum specimens were separated by centrifugation. Serum specimens were stored at −20°C at the hospital and then sent to Institut

Pasteur du Laos where they were stored at −80°C until use. HCV antibody testing was done by ELISA (Diasorin, https://www.diasorin.com) according to the manufacturer's instructions.

PCR and Sequencing

All HCV antibody positive samples were sent to the Luxembourg Institute of Health for reverse transcription PCR (RT-PCR) and sequencing. The diagnostic PCR was done by using the REALSTAR HCV RT-PCR KIT 2.0 (Altona Diagnostics, https://www.altona-diagnostics.com) according to the manufacturer's instructions. Amplification and sequencing of the core/E1 and NS5B regions of the HCV genome were performed by using previously described primers (*2*,*3*). MEGA version 7.0.14 (*4*) was used for the phylogenetic analyses and to calculate genetic distances between sequences.

Data Analysis

The data were described by descriptive and analytical biostatistics. Summary statistics were calculated. Bivariate and multivariable analyses were performed for the independent variables (sex, age, ethnicity, occupation, marital status, religion, place of birth, and diagnosis) and the dependent variable (HCV antibody seropositive). In bivariate analyses, the Chi2 test or the Fisher exact test were used as appropriate. Variables with a p value <0.2 in bivariate analysis were included in the multiple logistic regression model. Binary regression was conducted by using a stepwise method for removing variables one by one, and the Akaike Information Criterion of the model was calculated to determine the appropriate variables to include in the final model. A p value <0.05 was considered statistically significant. Statistical analyses were performed by using Stata version 14 (*5*). Maps were created using QGIS software version 3.4.14 (*6*).

Population Characteristics

Of the 753 participants selected for HCV antibody testing, the median age was 32 (range 5–90) years and 55.4% were females. They were from 275 villages; 33.7% of participants were from Samuoi district, 27.5% from Lao Ngarm, 25.2% from Saravan, 8.9% from Ta Oi, 3.3% from Toomlarn, 0.7% from Vapy, 0.3% from Khongxedone, and 0.4% from Lakhonepheng

districts. There were >9 ethnic groups represented, including Pako (35.2%), Lao (10.9%), Taoi (10.1%), and others. Most participants from Samuoi were of the Pako ethnicity (250/254 [98.4%], as compared with only 15/499 [3%] from the other districts). Most (62.9%) participants were married and the main occupations were farmers (60.4%) and students in primary, secondary, and further education (26.4%). Only 51 participants (6.8%) were in the hospital for hepatitis-related illness (acute hepatitis, fever, fever of unknown origin, or hepatocellular carcinoma).

References

1. Xaydalasouk K, Sayasinh K, Hübschen JM, Khounvisith V, Keomany S, Muller CP, et al. Age-stratified seroprevalence of vaccine-preventable infectious disease in Saravan, Southern Lao People's Democratic Republic. Int J Infect Dis. 2021;107:25–30. PubMed https://doi.org/10.1016/j.ijid.2021.04.033

2. Olinger CM, Jutavijittum P, Hübschen JM, Yousukh A, Samountry B, Thammavong T, et al. Possible new hepatitis B virus genotype, southeast Asia. Emerg Infect Dis. 2008;14:1777–80. PubMed https://doi.org/10.3201/eid1411.080437

3. Sandres-Sauné K, Deny P, Pasquier C, Thibaut V, Duverlie G, Izopet J. Determining hepatitis C genotype by analyzing the sequence of the NS5b region. J Virol Methods. 2003;109:187–93. PubMed https://doi.org/10.1016/S0166-0934(03)00070-3

4. Kumar S, Stecher G, Tamura K. MEGA7: Molecular Evolutionary Genetics Analysis version 7.0 for bigger datasets. Mol Biol Evol. 2016;33:1870–4. PubMed https://doi.org/10.1093/molbev/msw054

5. StataCorp. Stata Statistical Software: Release 14. College Station, TX: StataCorp LP; 2015.

6. QGIS.org. QGIS Geographic Information System. Open Source Geospatial Foundation Project [cited 2021 Jul 18]. http://qgis.org

7. Hübschen JM, Jutavijittum P, Thammavong T, Samountry B, Yousukh A, Toriyama K, et al. High genetic diversity including potential new subtypes of hepatitis C virus genotype 6 in Lao People's Democratic Republic. Clin Microbiol Infect. 2011;17:E30–4. PubMed https://doi.org/10.1111/j.1469-0691.2011.03665.x

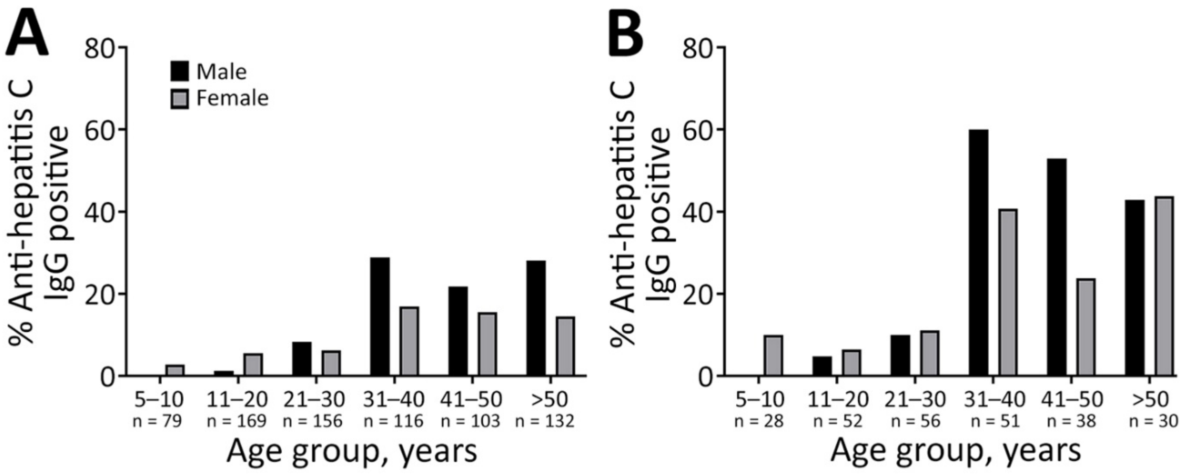

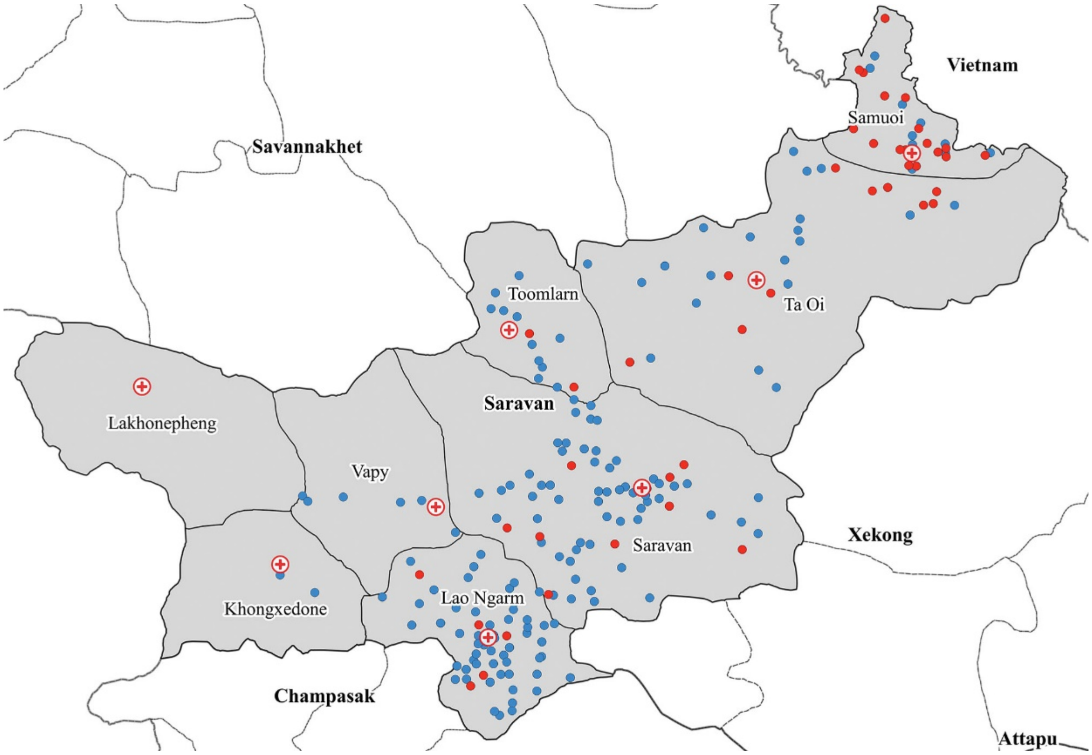

Appendix Figure 2. Location of villages with >1 hepatitis C virus antibody seropositive participant (red dots) and villages where all participants were hepatitis C virus antibody seronegative (blue dots). Participating hospitals are marked with a red cross. Participants were asked their place of residence, including village and district. The geolocation was determined according to data from the Lao National Statistics Bureau and plotted on QGIS (*6*).

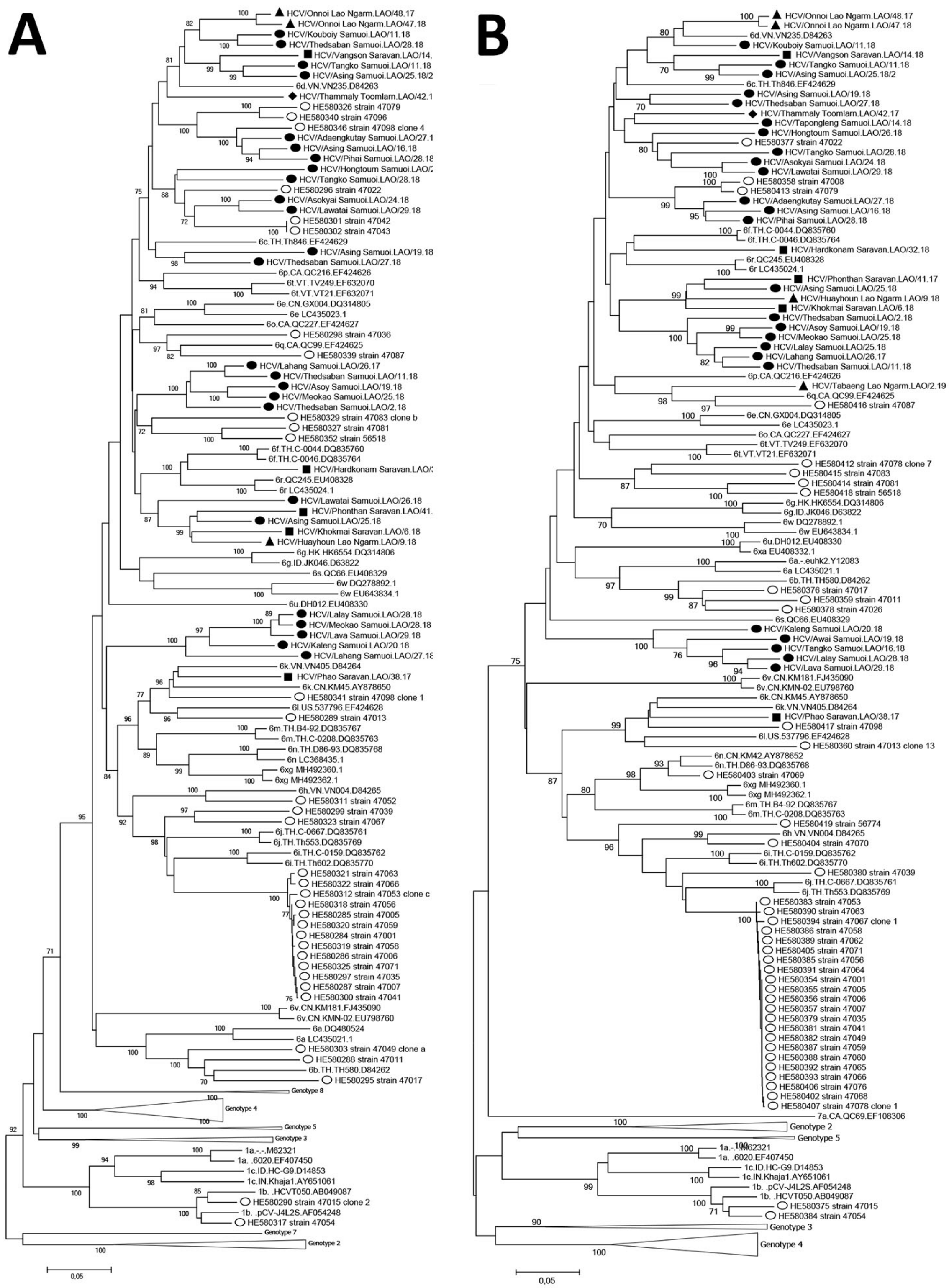

Appendix Figure 3. Phylogenetic trees based on A) 793 nt of the core/E1 gene and B) 340 nt of the NS5B region. The analyses were done with the Kimura 2-parameter model and the Neighbor-Joining algorithm. Only bootstrap values of ≥70 are shown. New sequences are marked with a black symbol,

previously published sequences from Lao People's Democratic Republic are marked with a white dot. The different shaped black symbols denote the different districts: dot, Samuoi; triangle, Lao Ngarm; square, Saravan; diamond, Toomlarn. Reference sequences are shown with the suggested subtype in front. There were no identical sequences and the genetic distances ranged from 2.3 to 33.6% in the core/E1 gene region and from 1.5 to 42.3% in the NS5B region. Most of the sequences formed new clusters and only a few clustered interspersed with previously reported sequences (*7*). Most of the sequences were from Samoi district, and they formed several different groups. Within individual clusters, mainly sequences from the same district and some from the same village grouped together.
